# Changing the tune using bioelectronics

**DOI:** 10.1186/s42234-021-00063-x

**Published:** 2021-02-23

**Authors:** Eric H. Chang

**Affiliations:** 1 Institute of Bioelectronic Medicine, The Feinstein Institutes for Medical Research, Northwell Health, 350 Community Drive, Manhasset, NY 11030 USA; 2 Donald and Barbara Zucker School of Medicine at Hofstra/Northwell, 500 Hofstra University, Hempstead, New York, 11030 USA

**Keywords:** Bioelectronic medicine, Neural interfaces, Peripheral nerves, Nanoclip

## Abstract

The desire to harness electricity for improving human health dates back at least two millennia. As electrical signals form the basis of communication within our nervous system, the ability to monitor, control, and precisely deliver electricity within our bodies holds great promise for treating disease. The nascent field of bioelectronic medicine capitalizes on this approach to improve human health, however, challenges remain in relating electrical nerve activity to physiological function. To overcome these challenges, we need more long-term studies on neural circuits where the nerve activity and physiological output is well-established. In this Letter, I highlight a recent study that takes just such an approach.

## Main text

In the late eighteenth century, the pioneering electrophysiologist Luigi Galvani showed that a jolt of electricity could make an isolated frog leg kick and twitch as if it was alive. This idea of “animal electricity” was one of the first demonstrations that electrical signals travel within the body to animate muscles. Galvani also believed that there was a natural form of bioelectricity produced within the body, possibly originating in the brain, to control organ function and muscle output (Piccolino 1997). While this was controversial in Galvani’s time, we now know that these posited bioelectrical signals are in fact nerve impulses that form the basis of communication throughout our nervous system. There is a vast and complex network of nerves that connect the brain to every vital organ and tissue within the body. The nervous system network provides an opportunity for scientists and clinicians to tap into this biological circuitry, either to eavesdrop on these electrical signals through nerve recording, or to intervene and manipulate them through neurostimulation. This approach describes the emerging field of bioelectronic medicine, which capitalizes on our ability to use nerve-interfacing devices to monitor and precisely control activity for the purpose of treating disease (Birmingham et al. 2014; Pavlov and Tracey 2019). This field is highly interdisciplinary, incorporating neuroscience, engineering, materials science, computing, and molecular medicine.

While advances in technology take place rapidly (think of Moore’s law), the true potential of bioelectronic medicine is maximized when it is applied to a biological circuit where the relationship between nerve activity and physiological function is well-understood. In this Letter, I highlight the recent work of Otchy and colleagues (Otchy et al. 2020) who provide a powerful example of applying advanced technologies to a circuit where the relationship between nerve activity and physiological output is robustly defined. Using microscale thin-film fabrication, electrophysiological recordings, and patterned neurostimulation to activate nerve fiber subsets, the authors show that high signal-to-noise ratio (SNR) interfaces can be achieved over multiple weeks on a nerve that is about the diameter of a single human hair. Studies like this define a new reference point for how to perform long-term interfacing with small peripheral nerves, and show how future clinical devices can exert tunable neuromodulation through patterned stimulation.

**Fig. 1:**
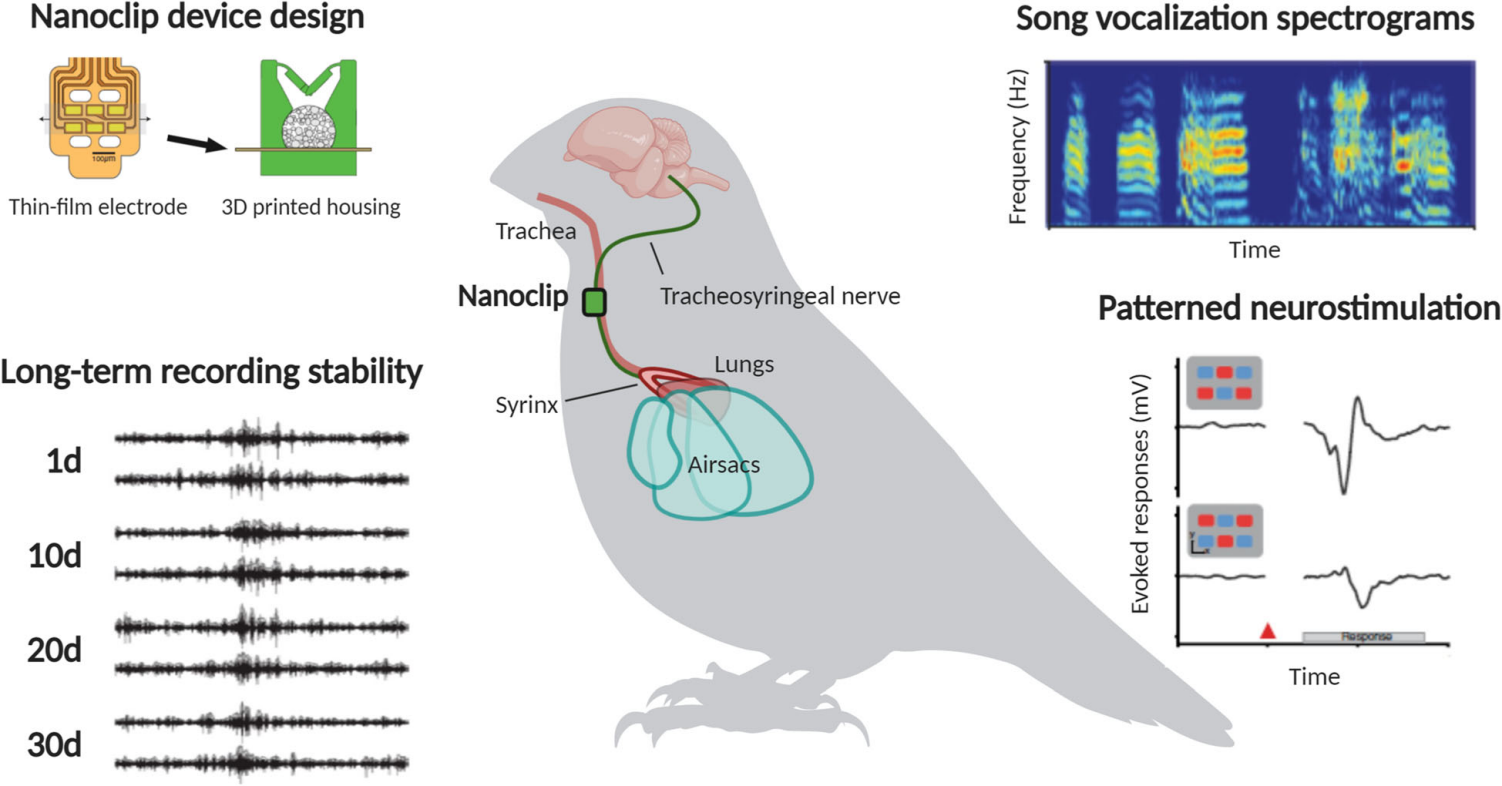
Optimized bioelectronic approach for peripheral nerve interfacing. Long-term recording and stimulation of the tracheosyringeal nerve in the zebra finch with an implanted nanoclip device. The study design exemplifies an optimized approach for performing recordings and tunable stimulation on a small peripheral nerve. The nanoclip device consists of a thin-film microfabricated electrode coupled to a custom 3D-printed housing with hinged trap doors to hold the nerve in place. High signal-to-noise ratio electrophysiological recordings were stable for over 4 weeks with the nanoclip. To measure functional differences in the output of the circuit, the acoustic structure of song vocalizations was quantified using high-resolution spectrograms. Patterned neurostimulation with current steering evoked distinct compound responses from the nerve reflecting differential fiber activation and also elicited distinct song vocalizations. Figure created using BioRender.com with portions adapted from (Otchy et al. 2020)

Otchy and colleagues used a custom device called the “nanoclip” that is capable of both electrical stimulation and recording on a 150-μm target nerve. The nanoclip, first reported by this research group in 2017 (Lissandrello et al. 2017), consists of a thin-film electrode with a 3D-printed housing that encapsulates the nerve with a hinged-door mechanism, thus minimizing torsion and potential damage to the nerve while ensuring a tight interface between the six gold pad electrodes and the nerve. In this study, they targeted the zebra finch tracheosyringeal nerve, which innervates the songbird vocal organ (the syrinx) and controls stereotyped song vocalizations. To demonstrate that the nanoclip could precisely modulate tracheosyringeal nerve activity and vocalization output over multiple weeks, they performed a number of experiments to show that: 1) recorded nerve activity was stable over 4 weeks, 2) songbird vocalizations could be precisely modulated and, 3) spatiotemporal patterning of neurostimulation successfully activated different subsets of fibers within the tracheosyringeal nerve bundle (Fig. 1).

Stability and specificity are critical features of any system designed for chronic implantation and neuromodulation. By modeling mechanical forces and body dynamics prior to device fabrication, the authors were able to design a small fit-to-nerve device that was easy to surgically implant and maintained stable, high SNR recording capabilities over at least 4 weeks. To demonstrate specificity, the authors used a technique called current steering where different parts of a multi-channel electrode array are turned on and off in a precise pattern to “steer” the current for a desired activation. By recording evoked compound action potentials through a second implanted nanoclip on the same nerve, they showed that current-steered stimulation modulated songbird vocalizations more precisely than bulk stimulation, and that different spatial patterns of stimulation reliably activated different subsets of nerve fibers. This is important because many peripheral nerves, such as the vagus nerve, contain several different calibers and types of fibers, each associated with different physiological functions (Ahmed et al. 2020). Therefore, the ability to achieve fiber-specificity through patterned neurostimulation works to maximize desired end-organ specific changes, while minimizing potential off-target side effects.

Studies like this demonstrate an important point, which is that multi-dimensional control of an output can only be achieved if we have a well-established understanding of the underlying neural circuit and how electrical activity relates to this output. By designing a paradigm where they could control the zebra finch vocalization system, the authors showed that evoked songbird vocalizations could be precisely and reliable controlled through nerve stimulation (Fig. 1). Similarly, without knowledge about the appropriate “transfer function” for a given neural circuit, we will struggle to design intelligent neuromodulation approaches for treating disease. So, if we hope to make an impact on human health through bioelectronic interventions, we need to synergize our evolving technological capabilities with basic science advances that inform us about mechanism. Other recent studies in this field give us a glimpse of what is possible, including work on a wireless, closed-loop optogenetic system for modulating bladder function (Mickle et al. 2019) and advances in the material sciences, such as conformable bioelectronics for interfacing with nerves and end organs (Fallegger et al. 2020). These developments will enable more seamless and flexible integration of bioelectronic interfaces with target tissues and provide the stability and specificity that is needed for precise, long-term neuromodulation.

Humans have been using electricity to treat ailments since the ancient Greeks and Romans, who used the electrical properties of torpedo fish to treat headaches and arthritis. While they did not know the mechanistic basis for the beneficial effects, they knew that the bioelectricity emitted by these fish relieved pain and reduced inflammation (Francis and Dingley 2015). Evidence that nerves were involved in transmitting the electricity came with Galvani’s frog experiments, which established the new science of electrophysiology. Now, more than two centuries later, precisely controlled pulses of electricity are being delivered via implanted vagus nerve stimulators in patients with medication-resistant epilepsy and depression (Labiner and Ahern 2007), as well as for rheumatoid arthritis and other inflammatory disorders (Koopman et al. 2016). Other invasive and noninvasive bioelectronic therapies are available to treat disorders ranging from sleep apnea to chronic pain. And while there are still gaps in our understanding of how electrical potentials relate to physiological outputs, contemporary tools and techniques are providing unprecedented access to the nervous system. This access, combined with increasingly sophisticated methods to measure and manipulate nerve activity, will allow us to finally harness the therapeutic potential of electricity to change the tune of nerve activity and to help treat human disease.

## Data Availability

Not applicable.

## Abbreviations

3D: Three-dimensional
SNR: Signal-to-noise ratio
μm: Micrometer
